# Analysis of the New Forming Process of Medical Screws with a Cylindrical Head of 316 LVM Steel

**DOI:** 10.3390/ma14040710

**Published:** 2021-02-03

**Authors:** Anna Dziubińska, Piotr Surdacki, Grzegorz Winiarski, Tomasz Bulzak, Krzysztof Majerski, Mariusz Piasta

**Affiliations:** 1 Mechanical Engineering Faculty, Lublin University of Technology, 36 Nadbystrzycka Str., 20-618 Lublin, Poland; piotr.surdacki@pollub.pl (P.S.); g.winiarski@pollub.pl (G.W.); t.bulzak@pollub.pl (T.B.); k.majerski@pollub.pl (K.M.); 2 Eurowkret Production Company, 28 Żakowiecka Str., 26-600 Radom, Poland; mariuszpiasta@eurowkret.pl

**Keywords:** medical screws, 316 LVM austenitic steel, metal forming, computer simulations, industrial research

## Abstract

The originality of this paper lies in the presentation of a new, innovative method for manufacturing medical screws with a cylindrical head of 316 LVM. This method is unique on a global scale, and its assumptions have been granted patent protection. The paper presents selected results of theoretical and experimental research on the developed process of forming of medical screws based on new technology. In the first part of the study a review of the types of screws used in the medical industry is made and the previous methods of their manufacture are described. The second part of the paper presents the assumptions and analysis of the elaborated process of metal forming of medical screws with a cylindrical head and ring thread made of 316 LVM austenitic steel. The theoretical analysis of the new process of forming a screw selected for testing was performed on the basis of numerical simulations. The experimental verification of the proposed theoretical solutions was carried out on the basis of laboratory tests, industrial research and qualitative research. The positive results obtained from computer simulations and experiments confirmed the effectiveness of the developed technology and the validity of its use in future in industrial practice.

## 1. Introduction

Austenitic steel of type 316 LVM (X2CrNiMo18-15-3) is characterised by high metallurgical purity and is dedicated to medical applications for implants [1]. This grade is a modified version 316 with low carbon, high nickel and molybdenum content [2,3]. The improvement of the chemical composition is aimed at maximising the material’s resistance to corrosion by obtaining a ferrite-free metallographic structure [4]. In order to ensure the purity and homogeneity of 316 LVM steel, a double vacuum induction melting + vacuum arc remelting process is generally used. This steel has good mechanical properties and corrosion resistance [5]. It has better mechanical properties after cold forming than hot forming [6,7]. Hot forming, including forging and all subsequent hot forming procedures for austenitic stainless steel ought to be in the temperature range of 930–1250 °C [8,9]. Some recommendations give a hot-forming temperature range of 1150–1250 °C for initial procedures and 930–960 °C for final procedures. Hot forming below 930 °C should generally be avoided. It is particularly important for stainless steel to avoid heating in the temperature range of 450–850 °C during heating or cooling, which causes precipitation of chromium carbides in the structure and reduces corrosion resistance and increases the brittleness of steel [10]. If the cooling rate from the hot-forming process is sufficiently high, there is not any need for additional heat treatment consisting in supersaturation of steel. Austenitic stainless steel, after hot forming, achieve mechanical properties characteristic for the condition after supersaturation, i.e., tensile strength Rm of 590–800 MPa, yield strength Rp0.2 = 285 MPa and elongation A of approx. 40% [11].

Austenitic stainless steel obtains significantly higher mechanical properties after cold forming, where the appropriate degree of crushing allows to control the increase in mechanical properties with a decrease in forming properties [12,13]. The hardening through crushing the 316 LVM steel as a result of cold forming makes it possible to obtain mechanical properties (tensile strength Rm) at the strength classes +C700, +C850, +C1000 and +C1150. After cold forming with an appropriate degree of crush, 316 LVM steel obtains a tensile strength Rm of 860–1100 MPa, yield point Rp0.2 = 690 MPa and elongation A of approx. 12% [11].

**Figure 1:**
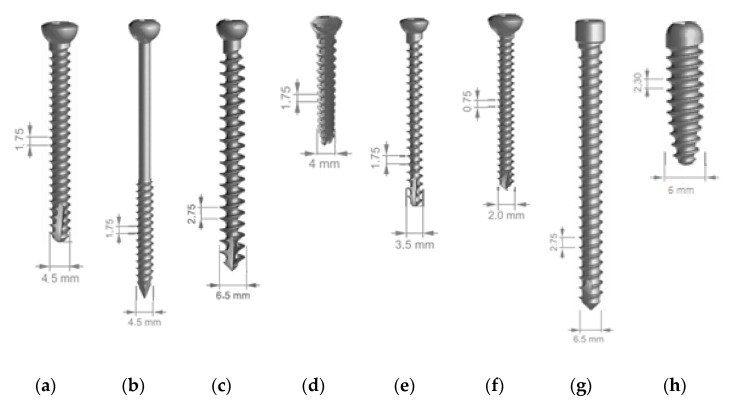
Types of bone screws made of 316 LVM steel by cavity treatment: cortical (**a**), cubic (**b**), spongy (**c**), cannulated (**d**), boat-shaped (**e**), for small bones (**f**), blocking (**g**), knee interference (**h**).

In medical applications 316 LVM steel is used for implantation into orthopaedic connecting wires, pins, skinning nails, bone nails, femoral heads, vertebrae, acetabulum bones, hip joints, knee joints, bones and nail plates, catheters, internal fastening devices, dental implants or staples [14,15,16,17]. In large quantities, 316 LVM steel is used in orthopaedics and traumatology for medical screws, including bone screws (Figure 1) [18,19,20]. This grade of stainless steel is also used in cardiovascular applications, such as guide wires or cardiac stents, and for surgical instruments: blood lancets, mandrels, trocars, etc. [9,13,21].

At present, medical screws are produced mainly by machining, additive processing and injection moulding methods [22].

The application of machining for the production of medical screws is described in the article [23,24]. The machining of a medical screw is to give the surface the desired shape, dimensions and surface quality by removing the material from the charge in the form of a cylinder using cutting tools. This technology is characterised by its high labour intensity, time consuming, energy-intensive process and the generation of high material losses often reaching up to approximately 50%. A screw produced by machining takes several minutes to several tens of minutes to produce, depending on the shape.

Among the methods of manufacturing medical screws, one can distinguish the additive processing described in the article [25,26]. In contrast to machining, the additive processing consists of applying and bonding successive layers of material until the finished medical screw is formed. The authors have described the method of manufacturing a medical screw with the SLM additive technology consisting in sintering successive layers of material powder with a concentrated laser energy beam. The advantage of this method is that it does not require the use of supporting structures in the printouts, which allows for printing more complex shapes. This method works well if you need to produce many screws in the shortest possible time, because the entire workspace is filled with models and produced all in one process. Other advantages are high process efficiency and high strength of the received screws. The screws produced in this way have a homogenous structure.

One of the ways of producing medical screws is also the injection moulding method developed by researchers from the Fraunhofer IFAM Institute for Production Engineering and Research of Applied Materials in Bremen. This technology involves the production of biodegradable medical screws made from polylactic acid, hydroxyapatite and medical stainless steel by injection moulding. Depending on the chemical composition of the screws, they can be biodegradable within 24 months. These screws can be manufactured precisely using conventional injection moulding methods, which means that there is no need for additional cavity processing. The resulting medical screws have similar properties to real bone. Their compressive strength is greater than 130 MPa, while a real bone can withstand 130–180 MPa.

Despite the existence of these technologies, new solutions are still being sought. Particularly noteworthy are the metal forming processes, which allow forming products with better mechanical and functional properties. Replacing the previously used technologies of medical screws production with the new methods of metal forming processing would allow forming products with better quality and at the same time reduce their manufacturing costs (reduced material and labour consumption).

It should be mentioned that the research subject matter undertaken is a response of the scientific world to the needs reported by medical producers for the development of an effective technology for the production of medical screws made of austenitic steel in terms of obtaining products of the highest quality. Therefore, the Lublin University of Technology has undertaken to develop a new method of forming medical screws using metal forming processes. This method is unique on a global scale, and its assumptions have been granted patent protection [27,28]. This paper presents assumptions and analysis of the new process of forming medical screws of steel with a cylindrical head. The positive results obtained from computer simulations, laboratory tests, industrial research and quality tests confirmed the effectiveness of the developed technology and the validity of its use in future in the production of screws made of 316 LVM steel used in the medical industry.

## 2. Methods and Materials

The material used for the test was 316 LVM (EN 1.4441; ISO 5832-1) austenitic steel in the annealed condition with the chemical composition and properties shown in Table 1 and Table 2, respectively. The steel was manufactured in the form of drawn wires with a diameter of 3.45 mm at the producer of metal biomaterials BHH Mikrohuta (Dąbrowa Górnicza, Poland).

**Figure 2:**
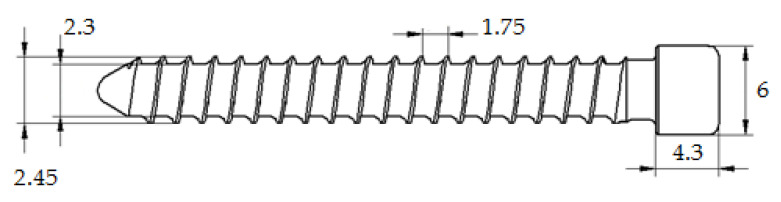
Medical screw with cylindrical head of 316 LVM steel used in medicine.

**Figure 3:**
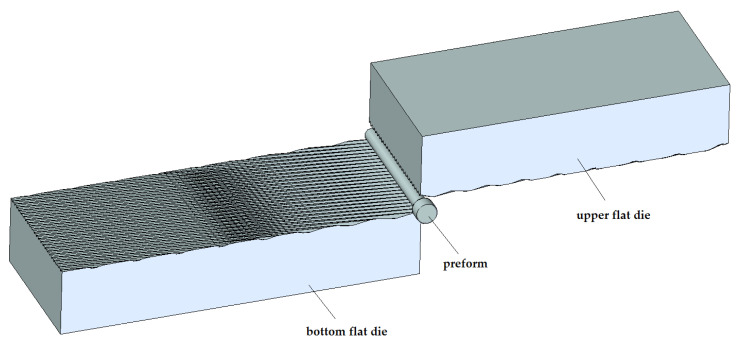
Geometric model of the screw ring thread rolling process.

**Figure 4:**
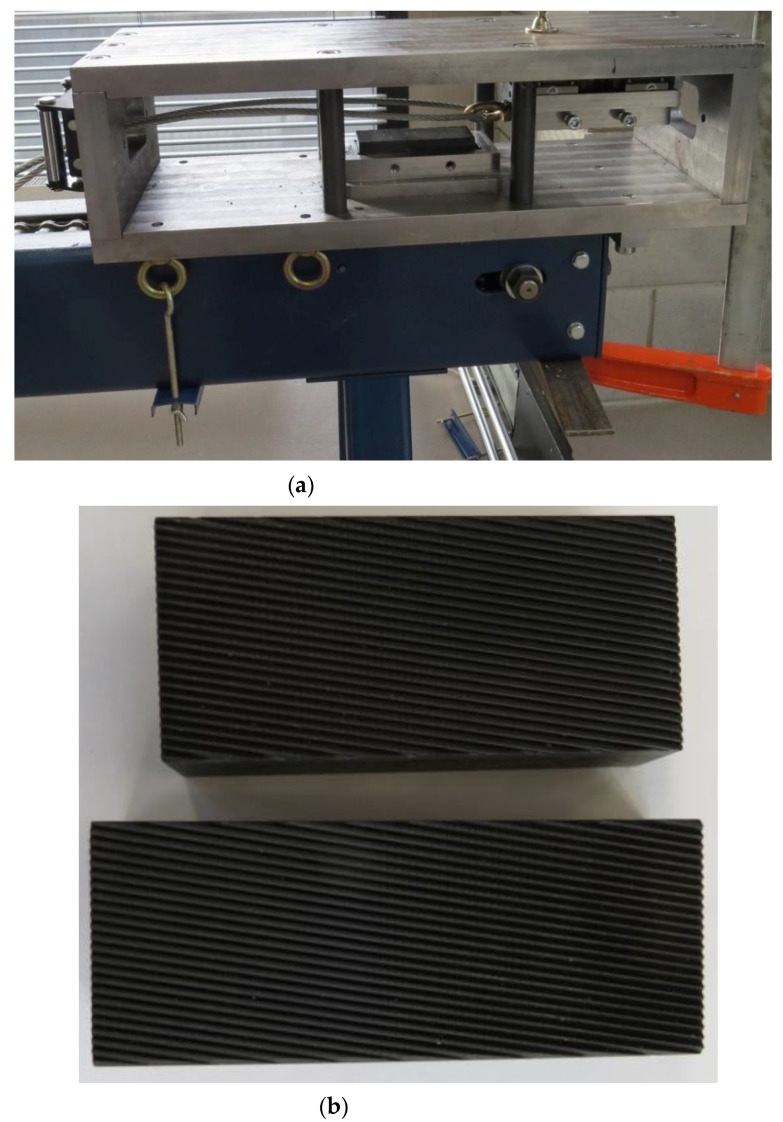
Device (**a**) and set of dies (**b**) for ring thread rolling of screw forgings.

**Figure 5:**
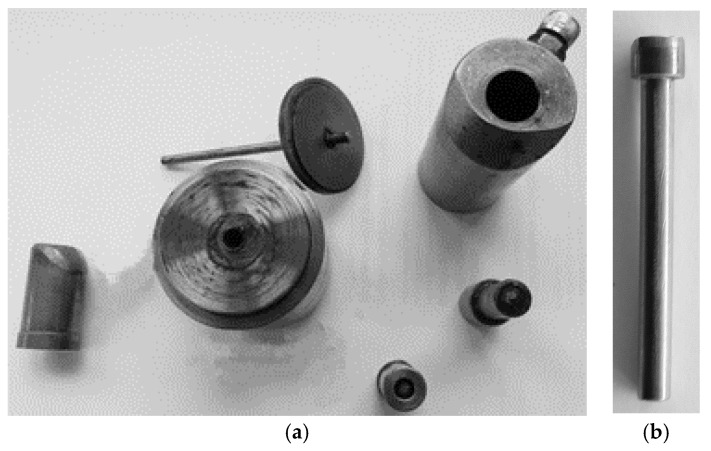
Set of dies for forging cylinder heads of medical screw forgings (**a**) and formed preform with a forged head of 316 LVM steel (**b**).

Figure 2 shows a medical screw with a cylindrical head with a ring thread selected for testing. Currently, these screws in the medical industry are manufactured by machining. The technology of cold forming of medical screws developed by the authors assumes operations of die head forging of the screw and cross-wedge rolling of the ring thread with flat dies. The theoretical verification of the assumed process of forming of medical screw forgings made of 316 LVM austenitic steel was performed on the basis of computer simulations. For numerical modelling of the process of die head forging of the screw, the program Deform 3D version 11 based on the finite element method (FEM) was used. The process of head forging was performed in cold conditions with lubrication in two operations. In the first operation, the end of the wire was upset. In the second operation of head forming, the screw punched the material into the die and filled the cylindrical shape. The simulations were performed under the assumption of a spatial deformation state using a full thermo-mechanical model. The billet was modelled as a rigid-plastic object divided into 100 thousand tetragonal elements. For the simulation a material model of 316 LVM steel was used, determined in own research. The speed of movement of the upper die was assumed according to the parameters of the machine used in the experiment, whose maximum speed is 740 mm/s. Friction on the material-tool contact surface was modelled as constant friction, for which the friction factor m = 0.2 was assumed. The simulations of the screw thread rolling were made in the Forge program version 8. For the purpose of numerical calculations, a geometrical model of the process was made in the program, as shown in Figure 3. The model consists of a billet in the form of a preform in the forged head and two tools in the form of flat dies with grooves with the outline corresponding to that of the ring thread of the screw being analysed, with the bottom die being immobile and the upper die making a moving motion at v = 100 m/s. Frictional conditions determined by Tresca’s friction model with a friction factor of 0.99 were used for the calculation. Based on the simulation results, the final shape of the test tools was determined. The tools for technological tests are shown in Figure 4b and Figure 5.

Experimental verification of the assumed process was performed in laboratory conditions in the laboratory of the Department of Computer Modelling and Metal Forming Technology at the Lublin University of Technology and in industrial conditions in the Eurowkret Production Company on industrial machines. In laboratory tests, a specially designed and manufactured thread rolling device (Figure 4a) and sets of dies (Figure 4b) were used for thread rolling, which were fixed on a laboratory drawing machine. Experimental tests of thread rolling in laboratory conditions were carried out for a billet in the form of preforms with a forged cylindrical head made of 316 LVM austenitic steel (Figure 5b) with identical forming conditions as those used in numerical simulations of the process. During the experimental research, the forming forces were also monitored.

Experimental verifications under industrial conditions at Eurowkret Production Company (Radom, Poland) were carried out on industrial machines i.e.,

- forging of screw heads was performed on a machine produced by the Zabierzów Machinery Factory’s (Zabierzów, Poland) model Pazm 6 using a set of dies shown in Figure 5a;
- thread rolling of the screw forgings was carried out on the Chun Zu CPR6S (Chun Zu Machinery Industry Co., Ltd, Kaohsiung, Taiwan) cross-wedge rolling machine using the dies shown in Figure 4b.

**Figure 6:**
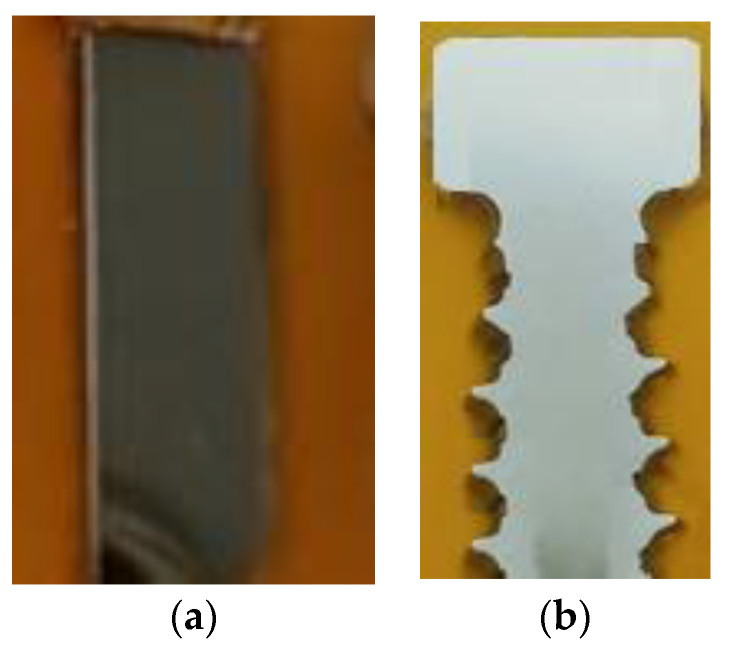
Metallographic specimens made on longitudinal cross-sections: (**a**) wire (raw material), (**b**) screws.

In order to analyse the correctness of the deformation process and to verify the simulation results, macro and microstructural tests were carried out, including a quantitative analysis of the microstructure of the products. Additionally, in order to check the degree of strengthening in selected areas, micro hardness tests were carried out using the Vickers method. Macro and microstructural analysis was performed for screw forgings and raw material with the use of optical microscopy (Nikon SMZ1500 stereoscopic microscope and Nikon MA200 metallographic microscope) (Tokyo, Japan) and the observations were made in a bright field. The metallographic specimens (Figure 6) for microstructural observations were prepared in the following stages:

- Making cuts on abrasive disc cutting machines and water cooling;
- Embedding preparations in the resin;
- Grinding on abrasive discs with grits 80, 220 with water cooling;
- Three-step polishing using a diamond suspension of 9 µm, 3 µm and colloidal silica 0.05 µm; and
- Electrolytic etching in a 10% oxalic acid solution, at 3V.

In selected areas of the samples where microstructures were analysed, microhardness tests were also performed using the Vickers method in accordance with PN-EN ISO 6507-1:2006. The tests were performed on the HV 0.5 scale using the Futuretech FM 800 microhardness meter (FUTURE-TECH CORP, Kawasaki, Japan). Additionally, in selected areas, grain size analysis was carried out in accordance with the ASTM E112 standard.

## 3. Results and Discussion

**Figure 7:**
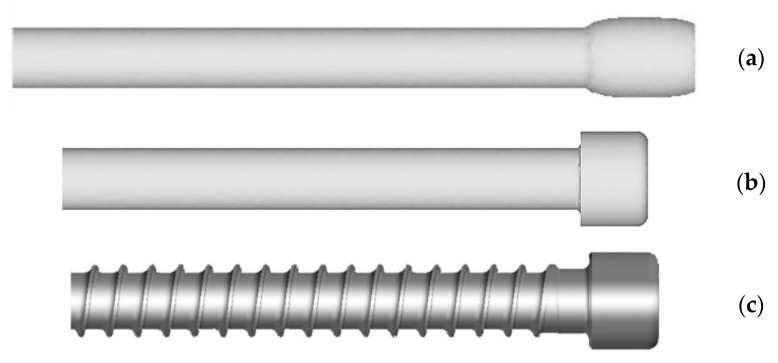
Geometry of the forging of a medical screw made of 316 LVM steel in individual stages of forming: (**a**) wire tip upsetting; (**b**) flashless forging of the head of the screw; (**c**) thread rolling.

The results of the simulation confirmed the possibility of metal forming of medical screw forgings according to the proposed technology. The Figure 7 shows the geometry of forging of medical screw form 316 LVM in individual stages of forming, i.e., 1st operation of the wire tip upsetting (Figure 7a), the 2nd operation of flashless forging of head of the screw (Figure 7b) and 3rd operations of cross-wedge rolling of the screw thread (Figure 7c). As can be seen in Figure 7b, the material filled the cylindrical impression of the die correctly and no errors were found in the formed head of preform. By also analysing the MES calculation, the thread shape of the screw can be found to be correct and reflects the ring thread of the screw shown in Figure 2.

**Figure 8:**
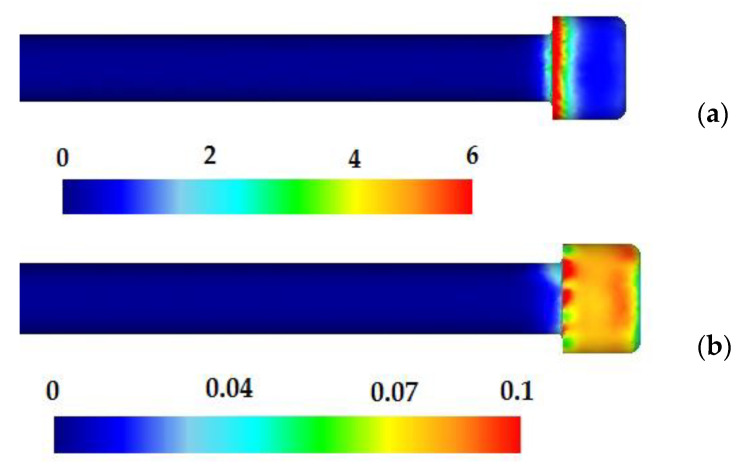
Distribution of the intensity of strain (**a**) and Cockroft–Latham damage parameter (**b**) in the preform of the screw.

On the basis of the simulation, more important information was obtained about the analysed process of forming of the forging of medical screw, including the distribution of the intensity of strains, stresses, or the criterion of destruction and forming forces. Figure 8a shows the distribution of intensity of strain in the formed cylindrical head of the screw. The numerical distribution of the intensity of strains in the formed preforms of screw is characterised by unevenness. The highest strains values are found on the radius between the front surface and the side wall of the screw head. The Cockroft–Latham criterion (C–L) [29] available in the Deform 3D program database was used to analyse the risk of cracks in the shaped screw head forging. Examples of distributions of the C–L integral value characterising this criterion in the formed preform are shown in Figure 8b. The results indicate that the highest values are found on the side walls of the cylindrical screw head.

**Figure 9:**
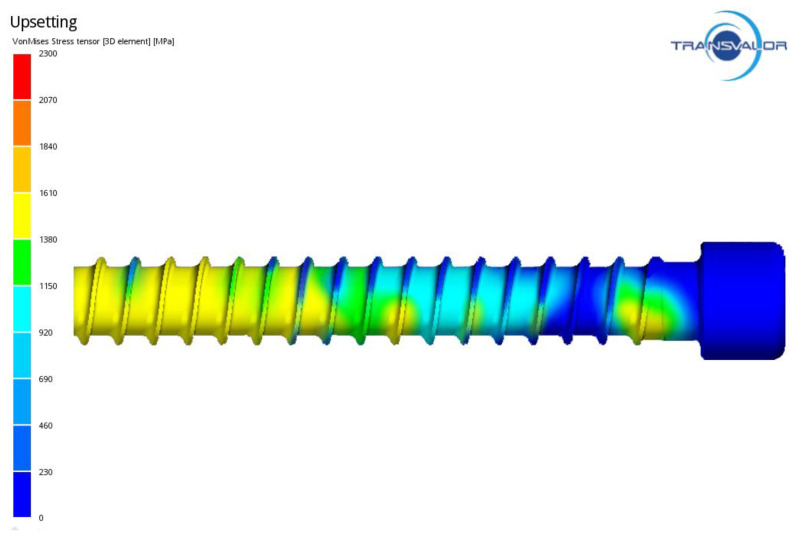
Distribution of stress in ring thread of the screw.

**Figure 10:**
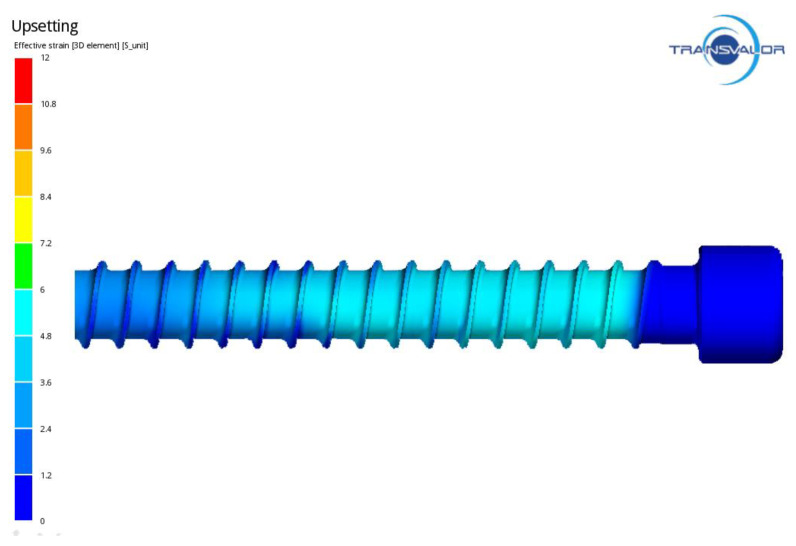
Distribution of intensity of strains in ring thread of the screw.

**Figure 11:**
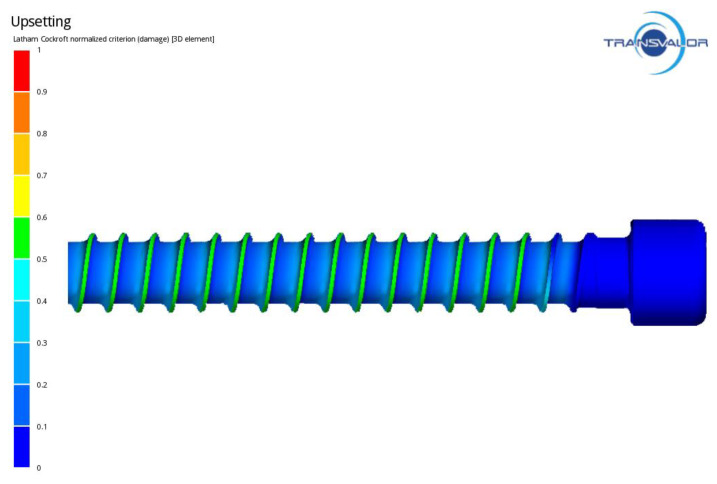
Distribution of damage in ring thread of the screw.

Figure 9 shows the distribution of stress in the formed ring thread of the screw, whose maximum values occur on the contact surface between the threading board and the screw. Figure 10 presents the distribution of intensity of strain on the screw surface, which is fairly homogeneous. The strain values during thread rolling reach a maximum of 6. Figure 11 shows an example of the MES calculation showing the areas exposed to loss of consistency according to C-L criterion in the formed thread. The highest values of the C-L integral are located on the thread coils, which indicates that there is the greatest risk of damage in these areas.

**Figure 12:**
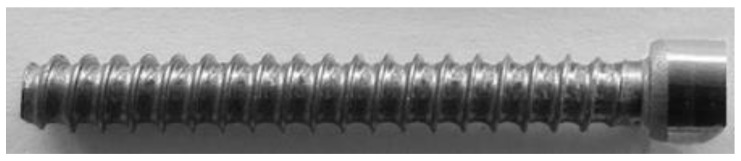
Forging of a screw with a ring thread of 316 LVM steel.

The results of the laboratory experiment and in real conditions in the Eurowkret Production Company confirmed the correctness of the modelled process of forming medical screw forgings. Figure 12 shows the forgings of a screw with a formed ring thread. A good correlation between the experiment and the simulation results was achieved.

**Figure 13:**
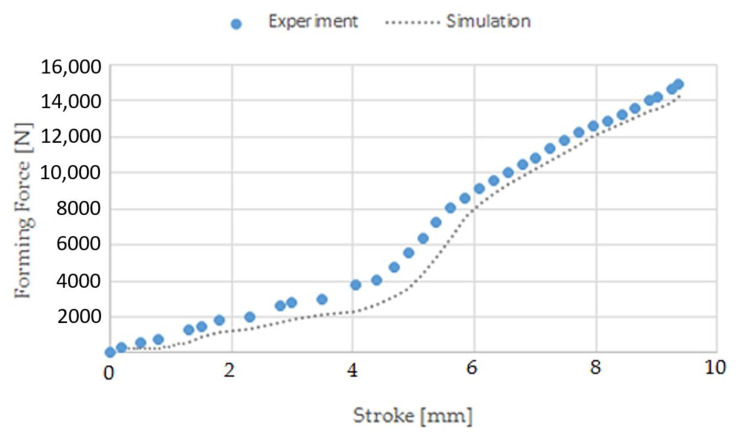
Forming force distributions during wire tip upsetting, measured experimentally and simulated.

**Figure 14:**
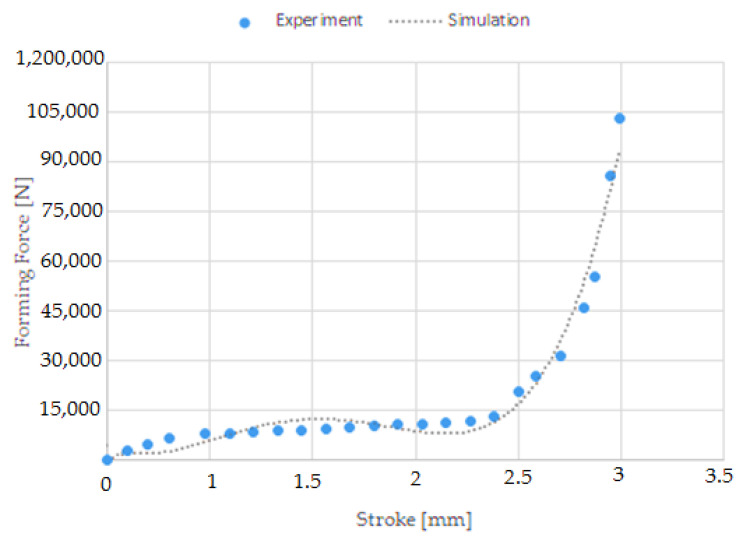
Forming force distributions during flashless forging of the head of the screw, measured experimentally and simulated.

**Figure 15:**
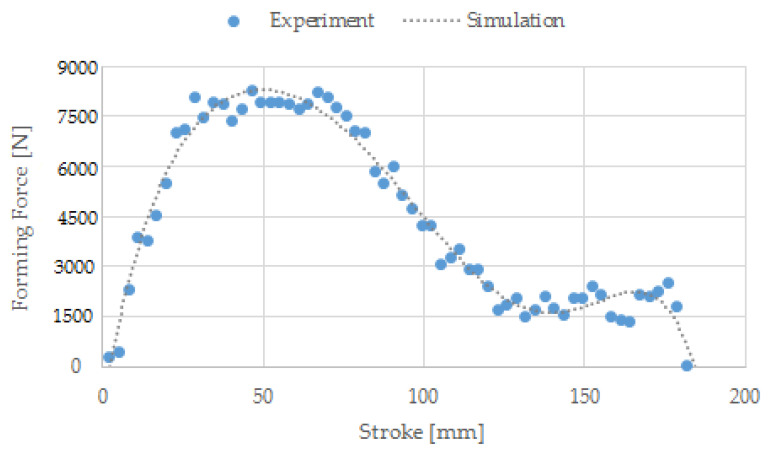
Forming force distributions during thread rolling, measured experimentally and simulated.

During the research, the forces needed to form the screw head and roll the screw thread were determined. A comparison of forces measured experimentally and simulated is shown in Figure 13, Figure 14 and Figure 15. Figure 13 shows the forming forces during wire tip upsetting, which increase until the set distance is reached. The maximum force was about 15,000 N for the experiment and about 14,000 N for the simulation. Analysing the force distribution, similar qualitative results were noted. Quantitatively, higher force values were noted during the experimental tests. The variation of the values is small and amounts to about 7%. Figure 14 shows the forming force distributions during flashless forging of the head of the screw. The forming force during the filling of the die blank increases until it reaches its maximum values, which are about 105,000 N for the experiment and about 95,000 N for the simulation. The distributions obtained from calculation and experiment are qualitatively similar. Figure 15 shows the forming force distributions during thread rolling, measured experimentally and simulated. During forming, the force increases until the tools move deep into the rolled bar by the value of the assumed crease and form the thread coil. Then, during calibration, the force decreases as the shape inaccuracies are removed, until the correct screw thread geometry is formed. The maximum force during thread rolling is 8250 N. The distributions of the forming forces from calculation and experiment are qualitatively and quantitatively similar.

Figure 16, Figure 17, Figure 18, Figure 19, Figure 20, Figure 21, Figure 22 and Figure 23 show the obtained microstructures in qualitative research.

**Figure 16:**
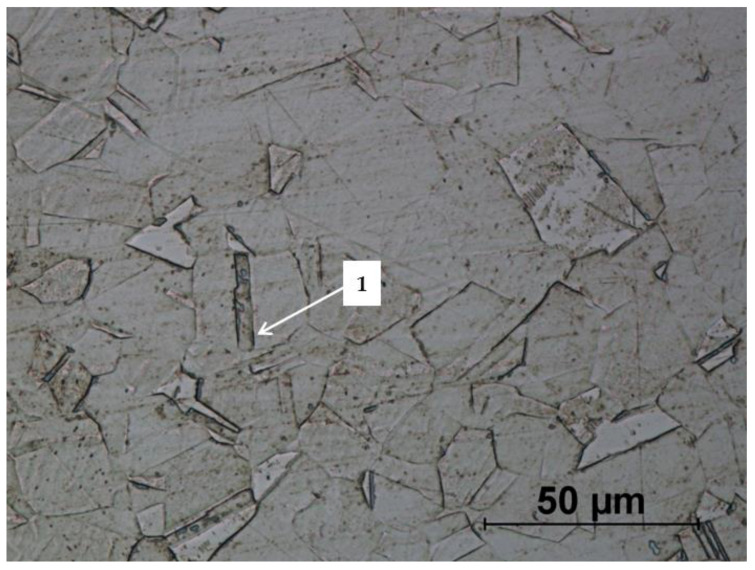
Microstructure of drawn wire (raw material): 1—annealing twins.

The microstructure of the drawn wire used to produce the screw (Figure 16) consists of equiaxed austenite grains. The annealing twins (1) are visible in the analysed area, which confirms the declared state of delivery (soft). The analysed microstructure is homogeneous in the whole section. The grain size is also typical for this type of wires and the average grain diameter is equal to 12.47 µm.

**Figure 17:**
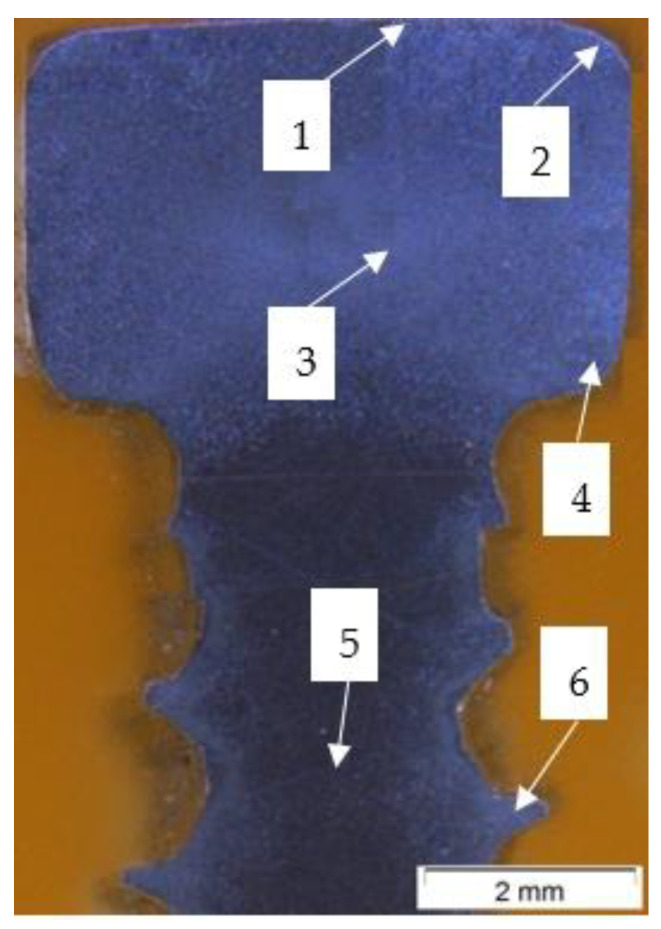
Cross-section of rolled screw after etching process: 1—the area of the frontal surface of the head; 2—the area of the radius between the frontal and side surfaces of the head, 3—central head area; 4—the area of the radius between the side surface and the bottom surface of the head; —the centre of the threaded part of the screw; 6—thread hump area.

On the basis of macrostructural observations of etching metallographic specimen (Figure 17), areas 1–6 were selected for microstructural studies of the screw.

**Figure 18:**
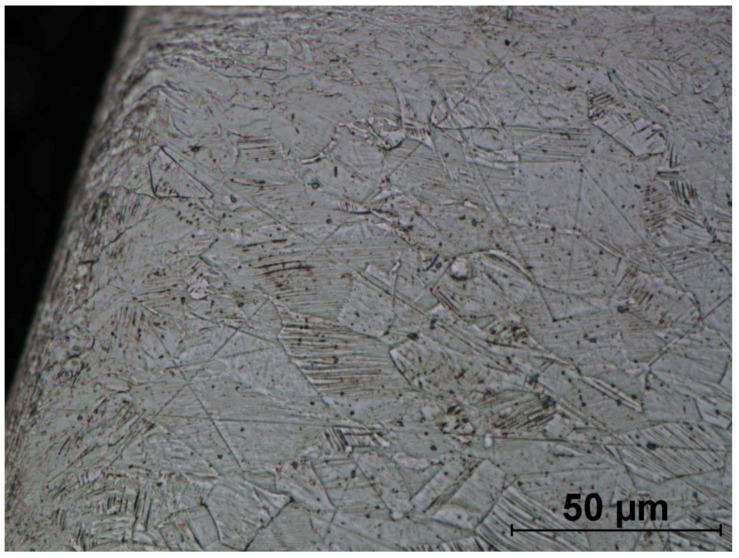
Screw microstructure, area 1.

**Figure 19:**
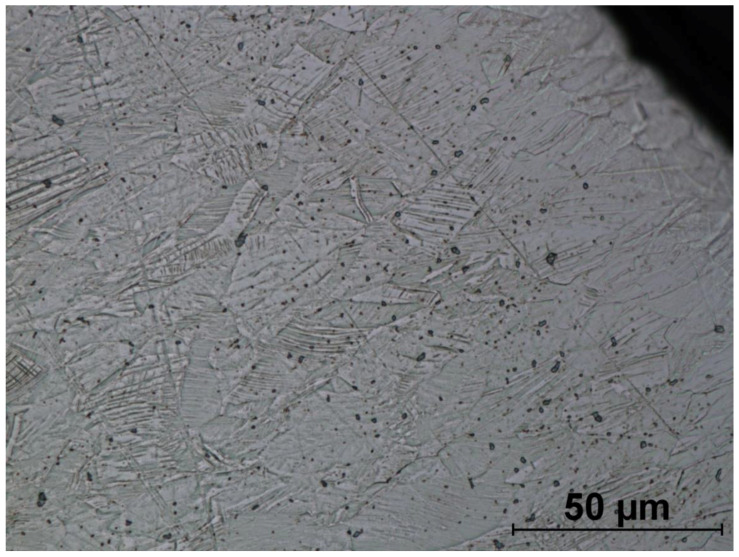
Screw microstructure, area 2.

**Figure 20:**
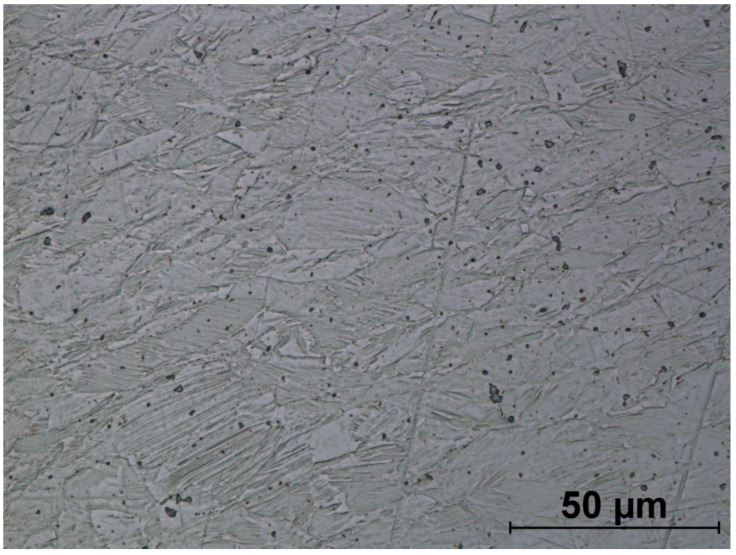
Screw microstructure, area 3.

**Figure 21:**
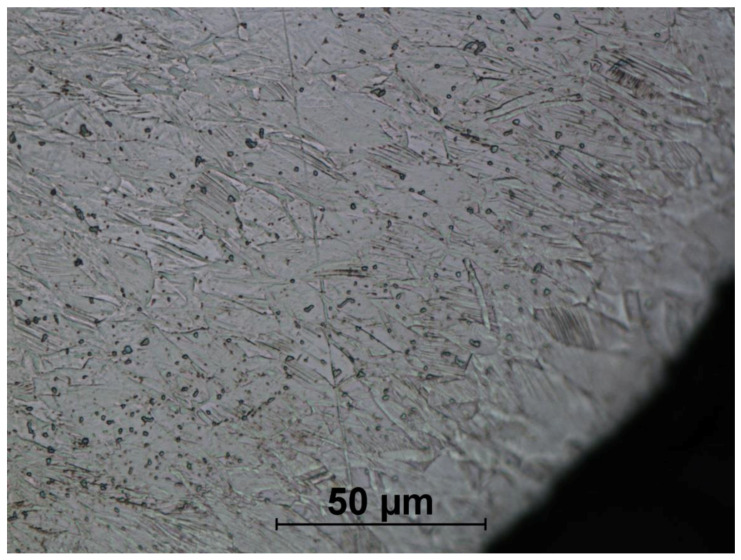
Screw microstructure, area 4.

**Figure 22:**
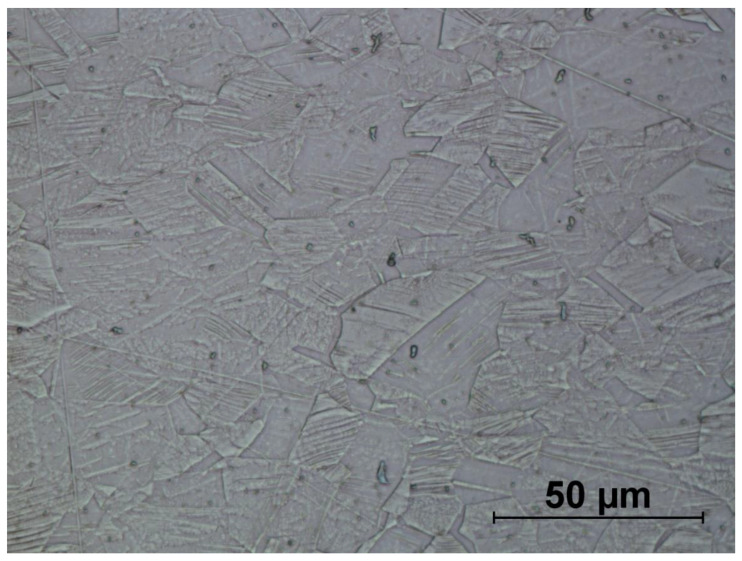
Screw microstructure, area 5.

**Figure 23:**
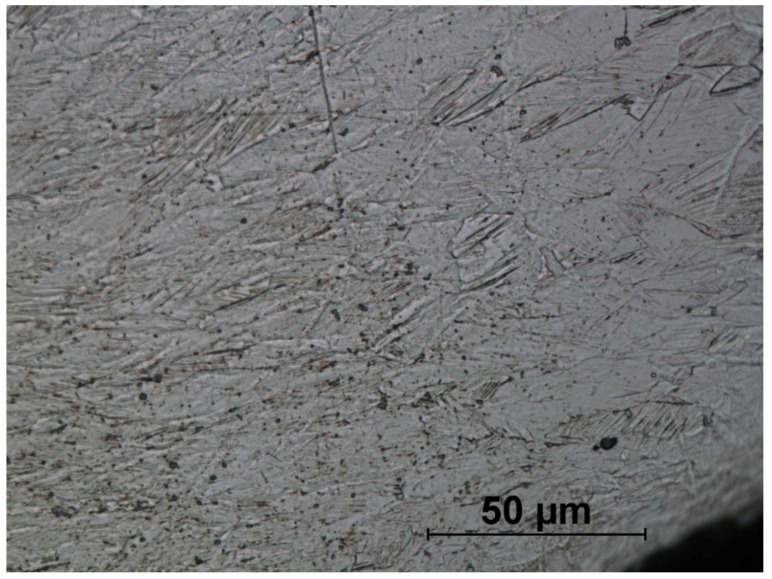
Screw microstructure, area 6.

The microstructures of formed screws shown in Figure 18, Figure 19, Figure 20, Figure 21, Figure 22 and Figure 23 are also austenitic. The entire cross-sectional areas of the screws show clear signs of deformation of the structure due to plastic deformation. The degree of structural changes is related to the location of the individual areas under analysis. For screw in the area of the head (areas 1–4) both at the surface and in the central part of the head, clear, densely located twin boundaries and slip bands due to deformation were observed. The accumulation of the deformation results in a reduction of the grain area, the size of which is not homogeneous. The average diameter of the grains in regions 1–4 is varied and belongs to the range from 7.91 to 10.42 µm. The core of the screw working part (area 5), is dominated by uniaxial grains, which indicates a low degree of deformation, however, a large number of twins indicates that the material has been plastically processed, but in a small extent. The average grain diameter is similar to that observed in the input material sample and is equal to 12.62 µm. In the thread hump zone (area 6), a structure analogous to (areas 1–4) was observed, revealing a large number of slip bands and significant deformation of the grains in the direction of material flow. The average grain diameter in area 6 is equal to 9.29 µm.

The microstructure of the analysed screw is characterised by considerable heterogeneity due to differences in plastic deformation values for individual areas. The structural changes are mainly related to the appearance of a significant number of twins, grains cut by the slip bands and deformations in the direction of the material flow.

It is therefore necessary to consider the use of appropriately selected post-rolled heat treatment processes in order to homogenise mechanical properties and ensure proper corrosion resistance.

The hardness measurements obtained in Table 3 indicate a significant increase in hardness caused by the process of head forging and rolling the screw thread. The value of the increase in relation to the input material is correlated with the degree of structural changes expressed in the average diameter of the grains in a given area (Table 3) and are also consistent with the simulation results. In the zone with the greatest structural changes, located at the bottom of screw head and on the thread hump of the rolled thread, the increase in hardness associated with the strengthening effect is greatest and amounts to over 100%.

## 4. Summary

This paper presents new possibilities of manufacturing medical screws using metal forming processes. On the basis of theoretical and experimental research, it has been found that in the assumed forming process it is possible to obtain forgings of medical screws with cylindrical head and ring threads of the correct shape. The advantage of the developed technology of metal forming screws over the hitherto applied machining lies in the following:

- Increasing the efficiency of product manufacturing;
- Reduction of production costs. This advantage results from lower material, time, labour and energy consumption of the new process. It is possible to reduce process waste by up to 45% compared to machining. A screw produced by machining takes several minutes to several tens of minutes to produce, depending on the shape. In the case of metal forming processes for producing screws: forging the screw head takes a few seconds and rolling the screw thread also takes a few seconds. Due to the reduction of the manufacturing time of the screws according to the new technology, this will result in a reduction of the labour and energy intensity of the process;
- Increase in product quality. Higher product quality is associated with a more favourable structure and high surface smoothness as a consequence of the application of plastic working processes, which results in better strength and service properties;
- Pro-environmentality. The new process’s environmental friendliness results from production that is less harmful to the environment, i.e., low waste by reducing material losses.

The advantage of the new technology is its versatility. This method can be used to form screws from various metal biomaterials.

The numerical analyses carried out confirm the advantages of using computer simulations when developing new technologies [30]. Thanks to them, multi-variant analysis is possible at the process design stage. It allows to analyse the kinematics of material flow in the new process, assess the quality of the obtained geometry of the product, analyse the process in terms of: state of stress, deformation, cracking, and more precisely plan experimental studies and reduce their costs. It also translates into a reduction of costs and time of implementing new technologies into production. Based on the results of the simulation, the final shape of prototype tools used for testing was also determined.

The laboratory and industrial tests carried out made it possible to determine the optimum technological parameters for the correct implementation of the process of metal forming of screw forgings and to produce technology demonstrators with qualitative tests. It should be mentioned that due to the adopted scheme of the process, the microstructure of the obtained screw forgings is characterized by heterogeneity related to the occurrence of different deformation values in individual areas of the product. The quantification of structural change expressed by increase in hardness as a result of hardening and the change in the average grain diameter confirm the results of numerical analyses in terms of the expected deformations in individual areas. It is, therefore, necessary to consider the application of appropriately selected heat treatment processes after the screw thread rolling operation in order to homogenize the structure and mechanical properties and ensure proper corrosion resistance.

## Figures and Tables

**Table 1:** Chemical composition of 316 LVM steel used in the experiment (wt%).

Material	C	Mn	Si	P	S	Cr	Ni	Mo	Fe
316 LVM	0.019	1.72	0.34	0.023	0.001	17.60	14.70	2.86	Balance

**Table 2:** Mechanical properties of 316 LVM steel.

Tensile Strength (MPa)	Yield Strength 0.2 (MPa)	Elongation A100 (%)
596	302	67.70

**Table 3:** Results of microhardness measurements and average grain diameter.

Sample	Measurement Number						Mean (HV)	Standard Deviation	Average Grain Diameter (µm)
	1	2	3	4	5	6			
Wire	156.3	157.2	156.7	161.4	158.3	156.2	157.7	1.8	12.47
Screw, area 1	319.3	295.3	289.5	291.3	302.6	295.3	298.9	10.0	10.42
Screw, area 3	323	346.8	331.7	333.2	329.5	338.9	333.9	7.5	9.87
Screw, area 4	353.4	352.1	649.6	355.6	354	350.8	352.6	2.0	7.91
Screw, area 5	238.4	247	241.1	248.3	243.2	245.3	243.9	3.4	12.62
Screw, area 6	340	293.4	332.6	355.4	282.9	326.4	321.8	25.6	9.29

## Data Availability

The data presented in this study are available on request from the corresponding author. The data are not publicly available due to protection of intellectual property.
